# Effects of Casein Phosphopeptide-Selenium Complex on the Immune Functions in Beagle Dogs

**DOI:** 10.3390/ani12162037

**Published:** 2022-08-10

**Authors:** Wencan Wang, Ling Xu, Yong Cao, Guo Liu, Qianru Lin, Xin Mao

**Affiliations:** 1 Chongqing Sweet Pet Products Co., Ltd., Chongqing 400000, China; 2 Guangdong Provincial Key Laboratory of Nutraceuticals and Functional Foods, College of Food Science, South China Agricultural University, Guangzhou 510642, China

**Keywords:** casein phosphopeptide–selenium complex (CPP-Se), immunity, lymphocytes, cytokines, dogs

## Abstract

**Simple Summary:**

Although the functions of casein phosphopeptide (CPP) and selenium (Se) as immunomodulators have been demonstrated, research on the regulation of pets’ immunity in the form of an organic combination of these two substances have yet to be reported, particularly in dogs. The purpose of this research was to look into the effects of the casein phosphopeptide-selenium complex (CPP-Se) on dog immunity. Our findings show that feeding a snack supplemented with CPP-Se for 30 days can increase the number of blood lymphocytes and improve the expression of cytokine-related genes in peripheral blood lymphocytes (PBL). In addition, serum levels of cytokine-related proteins and immunoglobulins were increased. Furthermore, cell proliferation and expression of cytokine-related mRNAs were also increased when PBL was exposed to CPP-Se in vitro. Collectively, feeding CPP-Se strengthens various immune indicators in dogs, thereby enhancing immunity. This research provides theoretical support for the development of related functional foods and formulations to improve the immunity and health of dogs and other pets in the future.

**Abstract:**

The health of pets is becoming a growing concern for the pet industry and its owners. Immunity is one of the foundational supports for health, thus developing a functional bioactive substance that can boost pets’ immunity is essential. Many studies have shown that casein phosphopeptide (CPP) and selenium (Se) can individually regulate immunity in many species, but there has been no reported research on the immunomodulatory function of casein phosphopeptide–selenium complex (CPP-Se). The objective of this study was to investigate the function of CPP-Se on immunomodulation in dogs. Twenty Beagle dogs were equally divided into two groups and fed either a control snack or a test snack supplemented with 0.03% CPP-Se for 30 days. Anticoagulated blood, serum and peripheral blood lymphocytes (PBL) were collected from dogs at 0 d, 10 d, 20 d and 30 d to detect the change in the number of immune cells and the expression of cytokine-related mRNAs and proteins. PBL isolated from blood were exposed to CPP-Se in vitro to measure the proliferative responses and cytokine-related mRNAs expression. During the time the test snack was fed, the number of lymphocytes increased significantly, whereas neutrophils and monocytes remained unaltered. The expression of interleukin-4 (*IL-4*), interleukin-6 (*IL-6*), tumor necrosis factor-α (*TNF-α*), interferon-γ (*IFN-γ*), CD4 molecule (*CD4*) and CD8α molecule (*CD8α*) was up-regulated, while interleukin-1β (*IL-1β*) was down-regulated, and interleukin-10 (*IL-10*) declined initially and subsequently increased. ELISA detection revealed a significant increment in serum IL-4, IL-6, Immunoglobulin M (IgM) and IFN-γ, except for IgG. Furthermore, CPP-Se treatment increased the proliferation and the expression of cytokine-related mRNAs in PBL cultured in vitro. This is the first study to demonstrate that CPP-Se can improve immunity in the dog.

## 1. Introduction

Pets, as a type of companion animal in intimate contact with humans, have a positive effect on human mental health, such as relieving neurological disorders, anxiety, depression, and also reducing loneliness in the elderly [1,2]. However, pets, on the other hand, are vectors of some pathogenic microorganisms, and antibiotic resistance caused and spread by pets creates a serious public safety risk [3,4,5]. As a result, there is a greater emphasis on disease resistance and wellness in pets, which ultimately benefits both their owners and society as a whole.

Immunity is the ability to resist disease, which can identify and eliminate foreign pathogenic factors, thereby making the organism free from disease. Until now, many studies have been conducted to improve the immunity of animals through the use of functional bioactive substances. Casein phosphopeptide (CPP) and selenium (Se) are commonly used as feed additives in a wide range of animals. CPP is the phosphorylated polypeptides derived from the proteolysis of casein. It was originally thought to promote calcium ion absorption [6], but its immunomodulatory function was later discovered in rabbits [7,8,9], pigs [10,11,12], mice [7,13,14,15,16] and humans [17,18,19,20]. These studies found that oral CPP can increase the production of immunoglobulins and stimulate lymphocyte proliferation. However, there have been no studies on the immunomodulatory function of CPP on dogs. Se is required by the body to perform antioxidant functions, but it also plays a vital role in immune regulation [21,22,23]. Se has been found to affect the content of serum inflammatory factors and antibodies, as well as the gene expression pattern of leukocytes [24,25,26]. In addition, Se deficiency or supplementation can alter the proliferation activity of dairy cow peripheral blood lymphocytes (PBL) [27,28]. Simultaneously, Se is involved in regulating the expression of immune genes in bovine support cells, improving sheep humoral and cellular immunity. Moreover, Se can also improve mice immune responses to vaccines [29,30,31]. Only a few studies on canine models have demonstrated the regulation of Se on immune functions. Kandil states that Se boosted the immune response in tapeworm-vaccinated dogs [32]. Lessard discovered that the serum obtained from dietary Se-deficient dogs inhibited the proliferation of autologous lymphocytes, thereby weakening the immune response [33].

Although the function of CPP or Se in enhancing animal immunity has been confirmed, the immunomodulatory function of the CPP-Se complex has not yet been tested in animals, especially in pets. Consequently, the goal of this study was to investigate the effects of CPP-Se on canine immunity, and we speculated that CPP-Se might also improve the immunity of dogs. To test the hypothesis, we fed dogs with a snack containing CPP-Se for 30 days and measured the number of blood immune cells as well as the gene and protein expression of cytokines in PBL and serum. Furthermore, we assessed the effects of CPP-Se on cell proliferation and cytokine-related mRNA expression of PBL cultured in vitro. We discovered that CPP-Se can improve various immune indicators in dogs, implying that it should be considered as a functional active substance in pet food development to enhance the immunity of dogs and other pets.

## 2. Materials and Methods

### 2.1. Preparation of Snack

The CPP-Se complex used in this experiment was provided by the College of Food Science, South China Agricultural University. We prepared a test snack with a CPP-Se content of 0.03% on the basis of the control snack (Chongqing Sweet Pet Products Co., Ltd., Chongqing, China). 

### 2.2. Animals and Study Design

In this study, twenty healthy beagle dogs (10 males and 10 females, 1-year-old, mean 12 kg) were equally divided into two groups. Each group includes five male and five female dogs, they are fed twice a day (9:00 a.m. and 15:00 p.m.) and provided adequate water. The following is fed to both the control and test group:

Control group: nutritionally complete and balanced staple (130 g) + control snack (30 g)

Test group: nutritionally complete and balanced staple (130 g) + test snack (30 g)

**Figure 1:**
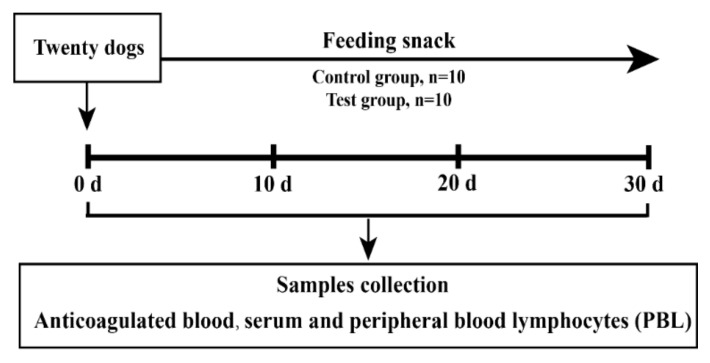
The schematic diagram of experimental design.

The staple was provided by Jiangsu Xietong Inc. (Nanjing, China; 25.95% protein, 4.88% fat, 3.32% fiber, 4037 ME Kcal/kg), and the snack feeding occurred 2 h after the staple feeding. This experiment lasted for 30 days, and the day before feeding was counted as day 0, all samples were collected on 10, 20 and 30 days, respectively. The body weight of all dogs was recorded before each sampling. All the experiments followed the institutional guidelines of South China Agriculture University. All the procedures were approved by the Institutional Animal Care and Use Committee of South China Agriculture University. The schematic diagram of the experimental design is shown in Figure 1.

### 2.3. Collection of Anticoagulated Blood and Serum

Blood was collected from the forelimb veins of the dogs in each group before feeding in the morning; 1 mL of blood was collected with EDTA-K_2_ anticoagulation for blood routine (Mindray, BC-2800, Shenzhen, China) analysis to detect the number of immune cells, including lymphocytes, monocytes and neutrophils. Meanwhile, the non-anticoagulated blood was collected according to the method of Zheng et al. [34]. Briefly, 2 mL blood was collected in a centrifuge tube without anticoagulant and after placing at room temperature for 1 h, and then centrifuged at 3000 rpm for 10 min at 4 °C, the serum was finally aliquoted into two EP tubes, one tube was used for biochemical analysis (Seamaty, SMT-120VP, Chengdu, China) and another one was stored at −20 °C for ELISA detection.

### 2.4. Isolation and Culture of Peripheral Blood Lymphocytes (PBL)

Peripheral blood lymphocytes (PBL) were isolated and cultured according to the methods described by Lee et al. [35] with some modifications. Before feeding in the morning, the canine PBL isolation kit (Tianjin Haoyang Biotechnology, Tianjin, China) was used to isolate PBL under sterile conditions. Briefly, 3 mL of venous peripheral blood was collected by EDTA-K_2_ anticoagulation. Then, 4 mL of separation liquid was pre-filled into a 15 mL centrifuge tube, and the blood was slowly added to the upper layer of the separation liquid along the tube wall and centrifuged at 2000 rpm for 25 min at room temperature. After centrifugation, the liquid was divided into four layers, the first layer is plasma, the second layer is lymphocytes, the third layer is separation liquid, and the fourth layer is erythrocytes. Then, the plasma was discarded and the lymphocytes were carefully transferred into a 15 mL centrifuge tube with a pipette tip, washed three times with a washing solution and finally, the RNA extraction lysate (Aidlab Biotechnologies, Beijing, China) was added to lymphocytes immediately for RNA extraction. In addition, a part of the lymphocytes from the control group was resuspended in RPMI-1640 media (Meilunbio, Dalian, China) containing 10% fetal bovine serum (FBS, PAN-Biotech, Aidenbach, Germany), 1% penicillin-streptomycin solution (Coolaber, Beijing, China) for subsequent in vitro culture.

### 2.5. PBL Purity and Viability Assay

The resuspended cells were centrifuged, and then the media was discarded and cells resuspended in PBS. A 20 μL cell suspension was used to prepare a cell smear, which was stained according to the Wright–Giemsa staining kit (Solarbio Life Science, Beijing, China), and counted under a microscope to identify cell morphology. In addition, 9 μL of cell suspension and 1 μL of 0.4% trypan blue staining solution were mixed (Tianjin Haoyang Biotechnology, Tianjin, China) and incubated at room temperature for 3 min, the number of living cells was counted under a microscope, and the lymphocyte viability was calculated.

### 2.6. Lymphocyte Proliferation Test

To evaluate whether CPP-Se has the ability to stimulate PBL proliferation in vitro, the cells were plated in 96-well plates and treated with CPP-Se at final concentrations of 0 (control), 0.5, 1, 2.5, 5, 10 and 20 μg/mL, while setting the blank group (only RPMI-1640 media). After 48 h of culture, 20 μL of CCK-8 (Coolaber, Beijing, China) solution was added to each well and culturing was continued for 4 h. The microplate reader detects the OD_450_ and the stimulation index was calculated according to the following formula to evaluate the optimal concentration of CPP-Se. According to the calculation results, in which CPP-Se treatment concentration has the highest stimulation index, the CPP-Se concentration is considered to be the optimal concentration.

$$\mathrm{Stimulation}\;\mathrm{index}\;(\%)=\frac{{\mathrm{OD}}_{450}\;\mathrm{of}\;\mathrm{test}\;\mathrm{group}\;-\;{\mathrm{OD}}_{450}\;\mathrm{of}\;\mathrm{blank}\;\mathrm{group}}{{\mathrm{OD}}_{450}\;\mathrm{of}\;\mathrm{control}\;\mathrm{group}\;-\;{\mathrm{OD}}_{450}\;\mathrm{of}\;\mathrm{blank}\;\mathrm{group}}\times 100\%$$

In addition, cells were treated with an optimal concentration of CPP-Se in 6-well plates for 48 h and harvested to detect relative expression of cytokine-related mRNAs.

### 2.7. Total RNA Extraction, cDNA Synthesis and RT-qPCR

Total RNA from lymphocytes was extracted with EASYspin Plus RNA Extraction Kit (Aidlab Biotechnologies, Beijing, China) according to the manufacturer’s instructions. The concentration and quality of total RNA were measured with a NanoPro 2010 (DHS Life Science and Technology, Tianjin, China). Reverse transcription of total RNA was performed by using ABScript III RT Master Mix (ABclonal, Wuhan, China) following the manufacturer’s protocol. All reverse transcription products were preserved at −20 °C. RT-qPCR was performed using 2× Universal SYBR Green Fast qPCR Mix (ABclonal, Wuhan, China) on the CFX 96 Real-Time PCR Detection System (Bioer Technology, Hangzhou, China). Briefly, RT-qPCR was carried out using 5 µL SYBR Green I Premix (ABclonal, Wuhan, China), 0.5 µL each of forward and reverse primers (Table 1), and 1 µL of cDNA, with the addition of RNase-free water to make a total volume of 10 µL. Thermal cycling was performed as follows: an initial step of denaturation at 95 °C for 3 min, 40 cycles of amplification at 95 °C for 5 s, and the primer-specific annealing temperatures were implied for 30 s. Relative expression levels of mRNA were determined using the 2^−∆∆^^CT^ method [36].

### 2.8. ELISA Detection of Cytokines

The contents of IL-4, IL-6, IgM, IgG and IFN-γ in the serum were measured with the corresponding enzyme-linked immunosorbent assay (ELISA) kits (FANKEL Industrial Co., Ltd., Shanghai, China). All operations were carried out in accordance with the corresponding instructions.

### 2.9. Statistical Analysis

The results are shown as mean ± SEM. All data were tested for normality and variance homogeneity prior to statistical analysis. The differences between the two groups were analyzed using the Student *t*-test. The evaluation of differences between multiple groups was performed using ANOVA followed by a Tukey posthoc test. Differences were considered significant when *p* < 0.05, differences were considered extremely significant when *p* < 0.01. 

## 3. Results

### 3.1. The Effects of CPP-Se on the Body Weight, Liver and Kidney Functions in Dogs

To assess whether CPP-Se feeding has adverse effects on the physiological functions of dogs, we performed serum biochemical analyses of all dogs fed for 30 days. We mainly focus on several indicators reflecting the physiological state of the body, including glutamyltransferase (GGT), alanine aminotransferase (ALT), aspartate aminotransferase (AST), total bilirubin (TB), serum creatinine (SCR), serum urea nitrogen (SUN) and inorganic phosphorus (PI). The results showed that these indicators of all dogs were within the normal range, suggesting no toxic effects were produced (Supplementary Table S1). Moreover, there was no significant change in the body weight of dogs before and after feeding CPP-Se (Supplementary Figure S1).

### 3.2. The Effects of CPP-Se on the Number of Blood Immune Cells in Dogs

**Figure 2:**
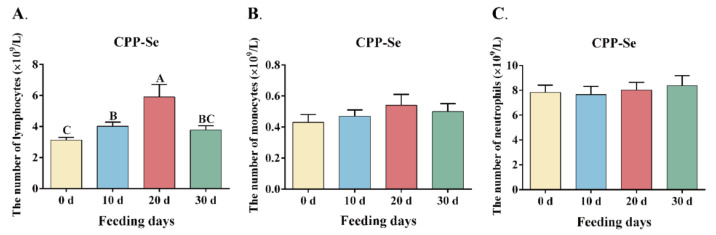
Changes in the number of lymphocytes (**A**), monocytes (**B**) and neutrophils (**C**) during the feeding period. Values are means with standard error bars, means with different letters are different (*p* < 0.01).

Routine blood analysis showed that the CPP-Se group had a significant increase in the number of blood lymphocytes on the 10 d (*p* < 0.05), 20 d (*p* < 0.05) and 30 d (*p* < 0.01) compared to the control group, while the number of monocytes and neutrophils did not change significantly (*p* > 0.05, Table 2). The number of lymphocytes in the CPP-Se group increased significantly on 10 d and 20 d (*p* < 0.01) compared to 0 d. Though the number of lymphocytes decreased significantly on 30 d (*p* < 0.01), the difference was not significant compared with 0 d (*p* > 0.05, Figure 2A). Furthermore, the number of monocytes and neutrophils was not significantly changed during the feeding period (*p* > 0.05, Figure 2B,C).

### 3.3. The Effects of CPP-Se on the Cytokine-Related mRNAs Expression of PBL in Dogs

**Figure 3:**
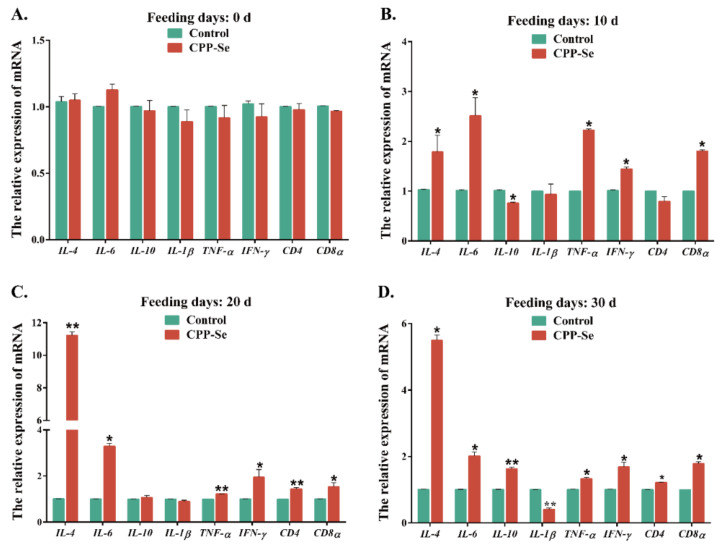
Changes in the expression level of cytokine-related genes in PBL of dogs fed 0 d (**A**), 10 d (**B**), 20 d (**C**) and 30 d (**D**). “*” indicates statistical significance at *p* < 0.05, “**” indicates statistical significance at *p* < 0.01.

RT-qPCR detection revealed the changes in the expression of cytokine-related genes in PBL of dogs during CPP-Se feeding. There was no significant difference in gene expression levels in dogs prior to feeding (*p* > 0.05, Figure 3A). On the 10 d, the expression level of *IL-4*, *IL-6*, *TNF-α*, *IFN-γ* and *CD8α* in the CPP-Se group was significantly higher than those in the control group (*p* < 0.05), while *IL-10* decreased significantly (*p* < 0.05), *IL-1β* and *CD4* did not change noticeably (*p* > 0.05, Figure 3B). Except for *IL-10* and *IL-1β*, which did not show significant differences at 20 d (*p* > 0.05), the expression level of other genes in the CPP-Se group was significantly higher than those in the control group (*p* < 0.05, Figure 3C). Only the expression level of *IL-1β* in the CPP-Se group was significantly lower than the control group at 30 d (*p* < 0.01), while the others were significantly higher (*p* < 0.05, Figure 3D).

### 3.4. The Effects of CPP-Se on the Content of Serum Cytokines and Immunoglobulins in Dogs

The levels of serum cytokines and immunoglobulins were measured by ELISA. The IgM level in the CPP-Se group fed on the 10 d did not differ significantly from the control group (*p* > 0.05). The content of IgM increased significantly (*p* < 0.01) after 20 d and 30 d of feeding, but there was no significant change in IgG during the feeding period (*p* > 0.05). The serum IL-6 in the CPP-Se group was significantly higher than that in the control group during the 10 d of feeding (*p* < 0.05), and this trend continued during the 20 d and 30 d (*p* < 0.01). On the 10 d (*p* < 0.01), 20 d (*p* < 0.01) and 30 d (*p* < 0.05), the CPP-Se group had significantly higher levels of IL-4 than the control group. In addition, when compared to the control group, the IFN-γ level of the CPP-Se group was not substantially different on the 10 d (*p* > 0.05), but increased considerably on the 20 d (*p* < 0.05) and 30 d (*p* < 0.01) of feedings (Table 3).

**Figure 4:**
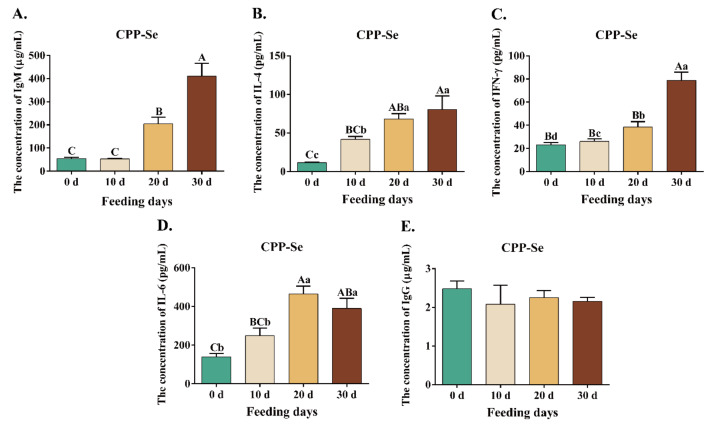
Changes in the content of IgM (**A**), IL-4 (**B**), IFN-γ (**C**), IL-6 (**D**) and IgG (**E**) during the feeding period. Different lowercase letters represent the level of significance at *p* < 0.05; different uppercase letters represent the level of significance at *p* < 0.01.

We compared cytokine content change at various time points. IgM, IL-4, and IFN-γ levels gradually increased with feeding time. Except for IgM on 10 d, these cytokines levels were significantly greater at all time points than on 0 d (*p* < 0.05). There was no significant change in IgG content (*p* > 0.05), and the level of IL-6 increased dramatically (*p* < 0.05), peaking on the 20 d and then declining on the 30 d (Figure 4). 

### 3.5. Identification of Lymphocytes

**Figure 5:**
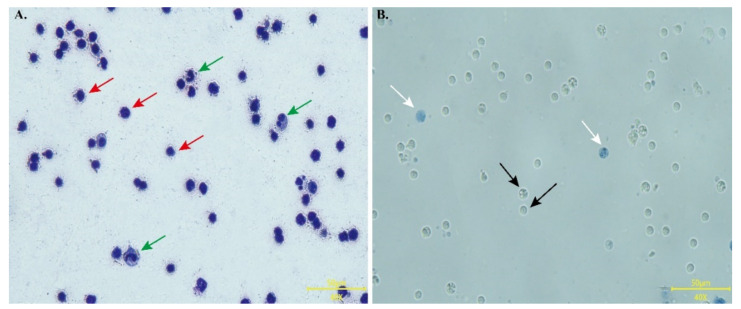
Morphology and viability identification of PBL. (**A**) Morphological characteristics of Wright-Giemsa stained PBL. Under the microscope, a small number of granulocytes (rod-shaped or lobulated nuclei, indicated by green arrows), and a large number of lymphocytes (round nuclei with little cytoplasm and light dark color, indicated by red arrows) were observed. (**B**) Viability detection of PBL using trypan blue staining. Live cells refuse to stain (black arrows) while dead cells are stained blue (white arrows). All pictures were screened at 400×, scale bar = 50 μm.

Prior to culture, lymphocytes extracted from peripheral blood were tested for purity and viability. Wright–Giemsa staining revealed that the lymphocyte nucleus was spherical, with intense staining, little cytoplasm, and purity > 90% (Figure 5A). Trypan blue can color dead cells blue, but it does not dye living cells. The statistical results show that lymphocyte viability was > 90% (Figure 5B). 

### 3.6. The Effects of CPP-Se on the Proliferation and Expression of Cytokine-Related mRNAs in Lymphocytes Cultured In Vitro 

**Figure 6:**
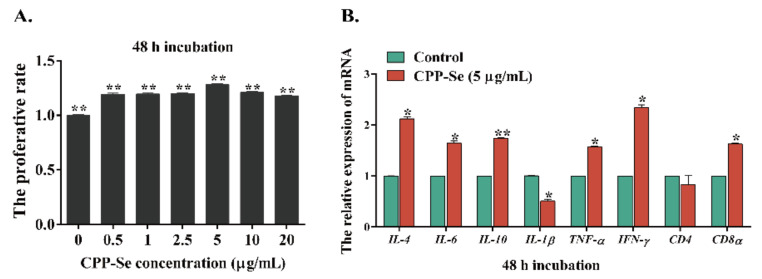
Changes in cell proliferation (**A**) and cytokine-related mRNAs expression (**B**) after PBL treated with 5 μg/mL CPP-Se in vitro. “*” indicates statistical significance at *p* < 0.05, “**” indicates statistical significance at *p* < 0.01.

Lymphocytes were treated with various concentrations of CPP-Se in vitro, and the cell proliferation rate was first determined. After 48 h of stimulation, cells treated with 5 μg/mL CPP-Se had the highest proliferation rate, which was significantly higher than the control group (*p* < 0.01, Figure 6A). As a result, lymphocytes were exposed to 5 μg/mL CPP-Se to detect the mRNA expression of cytokines. Our findings suggest that CPP-Se significantly increased the expression of *IL-4*, *IL-6*, *IL-10*, *TNF-α*, *IFN-γ* and *CD8α* genes in PBL (*p* < 0.05), and decreased the expression of *IL-*1β (*p* < 0.05), but there was no significant change in *CD4* gene expression (*p* > 0.05, Figure 6B).

## 4. Discussion

Currently, the regulation of animal immune functions by CPP or Se has been demonstrated in mouse models and other agricultural animals [11,13,15,37]. However, research on the immunomodulatory functions of the complexed form of CPP and Se has yet to be published in any animal, particularly in the pet dog. Therefore, our current study focuses for the first time on the immunomodulatory role of CPP-Se in a canine model.

The number of blood immune cells is a distinctly important indicator of the body’s immunity. Several studies have demonstrated that CPP and Se act as immunomodulators to promote lymphocyte proliferation [9,15,37,38,39]. In our study, dogs were fed a snack containing CPP-Se continuously for one month, and no physiological indicators were noticeably changed, such as body weight, liver and kidney function index. On the contrary, we found a significant increase in the number of blood lymphocytes in the dogs when compared to the control group. Moreover, Hata et al. found CPP only stimulated lymphocytes, not monocytes [9]. Our study also found that feeding CPP had no effect on the number of monocytes or neutrophils. These findings illustrate that CPP-Se may boost the immunity of dogs by increasing the number of lymphocytes.

Lymphocytes are non-proliferative under normal physiological conditions. Lymphocytes proliferate in response to external pathogens, allowing them to resist and eliminate pathogenic microorganisms. As a result, lymphocyte proliferation in vitro is an indicator for assessing the body’s immune function [40]. Studies by Tobita and Otani found that CPP has a mitogenic effect on mouse spleen lymphocytes to promote lymphocyte proliferation [13,15]. Hata et al. discovered that CPP can also promote the proliferation of mouse thymocytes and rabbit Peyer’s patch cells, indicating that CPP has mitogenic activity on lymphocytes [9]. Furthermore, Lessard et al. argued that Se was an essential substance in stimulating lymphocyte proliferation; the serum from diet Se-deficient dogs could inhibit lymphocyte proliferation in vitro [33]. In the present study, we found when the lymphocytes isolated from peripheral blood of dogs were exposed to 5 μg/mL of CPP-Se for 48 h in vitro, the cell proliferation rate was significantly increased, and the expression of cytokine-related mRNAs was significantly changed, which was consistent with the changes in the in vivo experiments, further indicating that CPP-Se has the ability to stimulate lymphocytes proliferation and cytokine expression, thereby enhancing the immunity in dogs.

Immunoglobulins and cytokines are a type of crucial physiological function regulator that the body produces in responses to infection, the immune response and inflammatory response, and they play a critical role in immune regulation [41,42]. IgG and IgM immunoglobulins provide defense for all tissues that blood reaches and aid in the prevention of blood-borne infections, sepsis, and the spread of microorganisms by neutralizing them as they enter the circulatory system [43]. Kitamura et al. fed CPP to sows one month before farrowing and discovered that the serum IgG level was significantly higher compared to the control group, and so was the milk IgG level on the day of farrowing. Furthermore, the serum IgG level in piglets was significantly increased after 10 days of lactation, indicating that CPP can increase IgG levels and promote immune response [11]. Interestingly, Otani et al. found that feeding CPP to piglets did not increase serum IgG and IgM levels [10]. Se also promotes the secretion of immunoglobulin. Swecker et al. discovered that Se supplementation in dairy cows increased serum IgG levels in colostrum and calves, but had no effect on IgM content [44]. Supplementation of an appropriate amount of Se promoted the ability of calves to synthesize IgM in lymphocytes cultured in vitro [45]. Liu and Li et al. found that Se could increase serum IgG content in pigs [24,25]. Our study found that IgG content did not change significantly during CPP-Se feeding, while IgM levels increased significantly, suggesting that CPP-Se may increase the canine immune response by regulating the synthesis and secretion of IgM through a different mechanism.

CPP has been shown to stimulate the expression of cytokines. Cytokines usually have pro-inflammatory or anti-inflammatory effects, and the balance between the two determines the direction of the inflammatory response. Major pro-inflammatory factors include *IL-6*, *TNF-α* and *IFN-γ*, and major anti-inflammatory factors include *IL-4* and *IL-10*. Among them, although *IL-4* is considered to mainly play an anti-inflammatory function, it also has a pro-inflammatory function [46,47]. Otani et al. discovered that oral administration of CPP could increase IL-6 levels in splenocytes cultured in vitro [14]; CPP had an effect on the mRNAs expression of cytokines, such as *IL-6*, *TNF-α* and *IL-1β*, in cultured bovine spleen CD19^+^ cells and human Caco-2 cells [13,20]. We discovered that feeding CPP-Se could significantly increase the gene expression of *IL-4*, *IL-10*, *IL-6*, *TNF-α* and *IFN-γ* in PBL, but *IL-1β* decreased significantly. Simultaneously, serum IL-4, IL-6 and IFN-γ protein levels increased significantly. In vitro treatment of lymphocytes with CPP-Se resulted in a trend of cytokine-related mRNAs expression similar to the in vivo experiments. Moreover, research found that IL-4 regulated antibody synthesis, hematopoiesis and inflammation, as well as the generation of effector T cell responses in vitro, which can induce B cells to release IgM [48,49]. IL-6 promoted the development of B cells into immunoglobulin-secreting cells, resulting in an increase in IgM content [50]. In light of the fact that CPP-Se dramatically raised serum IgM content of dogs, our findings suggest that CPP-Se may act as a regulator of cytokines and immunoglobulins levels to enhance canine immunity. Furthermore, we found a significant increase in the expression of *CD4* and *CD8α* mRNA. *CD4* and *CD8α* genes are involved in a variety of immune functions, including T cell activation, differentiation, interleukin-mediated pathways, and inflammatory signaling pathways [51,52,53,54,55]. Day noted that the decreased proportion of CD4^+^ cells in elderly dogs is associated with impaired immunity [56]. CD4^+^ and CD8^+^ cells also have antiviral properties and are involved in the healing of benign warts caused by low-risk papillomaviruses in animals [57]. In dogs, CD4^+^/CD8^+^ cells are critical against parasite proliferation; both demodex and atopic dermatitis are associated with CD4^+^/CD8^+^ cells [58,59,60,61]. In addition, Cortese et al. found that the number of CD4^+^ and CD8^+^ cells was significantly changed in dogs naturally infected by Leishmania infantum [62]. Thus, our findings suggest that CPP-Se may protect dogs from parasitic infections, but further research is needed to confirm this. 

## 5. Conclusions

Overall, our study found that CPP-Se has an effect on immunological markers in dogs. To begin with, CPP-Se treatment showed no signs of toxicity appeared in this study. Feeding a snack containing CPP-Se can efficiently increase the number of lymphocytes and the expression of cytokine-related mRNAs, as well as the concentrations of serum cytokines and immunoglobulins. Furthermore, in vitro investigations show that CPP-Se can stimulate lymphocyte proliferation and change the expression of cytokine-related mRNAs. To summarize, we demonstrated in vivo and in vitro that CPP-Se possesses immune-enhancing properties in dogs, implying it could be considered as a food additive in the future to enhance the immune response of dogs or other pets.

## Figures and Tables

**Table 1:** The primers of mRNAs for RT-qPCR.

Gene Name	Primer Sequences (5′→3′)	Tm (°C)	Size (bp)	Accession ID
*GAPDH*	F: GTCCCCACCCCCAATGTATC	60.0	128	NM_001003142
	R: GTGTAGCCCAGGATGCCTTT			
*IL-4*	F: GCTCCAAAGAACACAAGCGA	60.0	136	NM_001003159
	R: AGGTCTTGTTTGCCATGCTG			
*IL-6*	F: TCCTGGTGATGGCTACTGCTT	60.0	78	NM_001003301
	R: GACTATTTGAAGTGGCATCATCCTT			
*IL-10*	F: ACCACGACCCAGACATCAAG	60.0	127	NM_001003077
	R: TCCACCGCCTTGCTCTTATT			
*IL-1β*	F: ATATGAGCTTCGGGCTCTCC	60.0	174	NM_001037971
	R: TGTAGGGTGGGCTTTCCATC			
*TNF-α*	F: ACCTACTCTCTGCCATCAAGAGC	60.0	122	NM_001003244
	R: GAGTCGATCACCCTTCTCCAGT			
*IFN-γ*	F: TCCAGCGCAAGGCGATAAAT	60.0	106	NM_001003174
	R: CTGCGGCCTCGAAACAGATT			
*CD4*	F: CCAGAGCCAGAAGACAGTGG	60.0	190	NM_001003252
	R: GATCCAGAGCAAGGACGAGG			
*CD8α*	F: AAGTGGGTTAGACTTCGCCTG	60.0	119	NM_001002935
	R: CACGTCTTCTGTTCCTGTGGT			

**Table 2:** Effects of CPP-Se on the number of blood immune cells in dogs.

Number (×10^9^/L)	Groups	Feeding Days			
		0 d	10 d	20 d	30 d
Lymphocytes	Control	2.94 ± 0.15	2.87 ± 0.23 a	2.41 ± 0.27 a	2.38 ± 0.32 A
	CPP-Se	3.13 ± 0.17	4.02 ± 0.27 b	5.90 ± 0.81 b	3.78 ± 0.27 B
Monocytes	Control	0.46 ± 0.03	0.41 ± 0.05	0.46 ± 0.04	0.48 ± 0.04
	CPP-Se	0.43 ± 0.05	0.47 ± 0.04	0.54 ± 0.07	0.50 ± 0.05
Neutrophils	Control	7.71 ± 0.19	7.20 ± 0.41	7.37 ± 0.40	7.38 ± 0.46
	CPP-Se	7.83 ± 0.59	7.65 ± 0.67	8.02 ± 0.62	8.38 ± 0.80

Note: Means with letters are different from initial. Different letters in the same column represent the level of significance (lowercase letters at *p* < 0.05; uppercase letters at *p* < 0.01).

**Table 3:** Effects of CPP-Se on the levels of serum cytokines and immunoglobulins in dogs.

Content	Groups	Feeding Days			
		0 d	10 d	20 d	30 d
IgM (μg/mL)	Control	50.79 ± 6.06	51.30 ± 7.23	49.73 ± 3.66 A	54.65 ± 4.07 A
	CPP-Se	54.16 ± 6.09	53.14 ± 1.99	204.32 ± 29.14 B	411.24 ± 55.35 B
IgG (μg/mL)	Control	2.51 ± 0.17	2.40 ± 0.17	2.48 ± 0.11	2.19 ± 0.11
	CPP-Se	2.48 ± 0.20	2.08 ± 0.49	2.25 ± 0.18	2.15 ± 0.11
IL-6 (pg/mL)	Control	131.81 ± 2.71	126.59 ± 7.44 a	133.28 ± 7.84 A	132.62 ± 9.35 A
	CPP-Se	139.18 ± 17.67	249.98 ± 38.16 b	464.71 ± 40.88 B	389.84 ± 52.81 B
IL-4 (pg/mL)	Control	11.29 ± 0.73	12.99 ± 2.44 A	15.41 ± 4.21 A	12.97 ± 1.52 a
	CPP-Se	11.61 ± 0.72	41.73 ± 3.76 B	68.11 ± 6.77 B	80.48 ± 17.56 b
IFN-γ (pg/mL)	Control	23.35 ± 1.74	23.84 ± 1.40	23.68 ± 1.60 a	24.13 ± 2.16 A
	CPP-Se	23.17 ± 2.05	26.06 ± 2.25	38.34 ± 4.78 b	78.88 ± 7.07 B

Note: Means with letters are different from initial. Different letters in the same column represent the level of significance (lowercase letters at *p* < 0.05; uppercase letters at *p* < 0.01).

## Data Availability

Data are contained within the article.

## Supplementary Materials

The following supporting information can be downloaded at: https://www.mdpi.com/article/10.3390/ani12162037/s1, Figure S1: The body weight of dogs during the experiment; Table S1: Serum biochemical data of dogs fed for 30 days.
