# Supplementary material for: Effects of Casein Phosphopeptide-Selenium Complex on the Immune Functions in Beagle Dogs
Source: Animals (Basel). 2022 Aug 10;12(16):2037. doi: 10.3390/ani12162037 (PMC9404450; doi:10.3390/ani12162037)
Supplement: Supplementary file 1 [file animals-12-02037-s001.zip › animals-1831470-supplementary.pdf]

Supplementary Materials

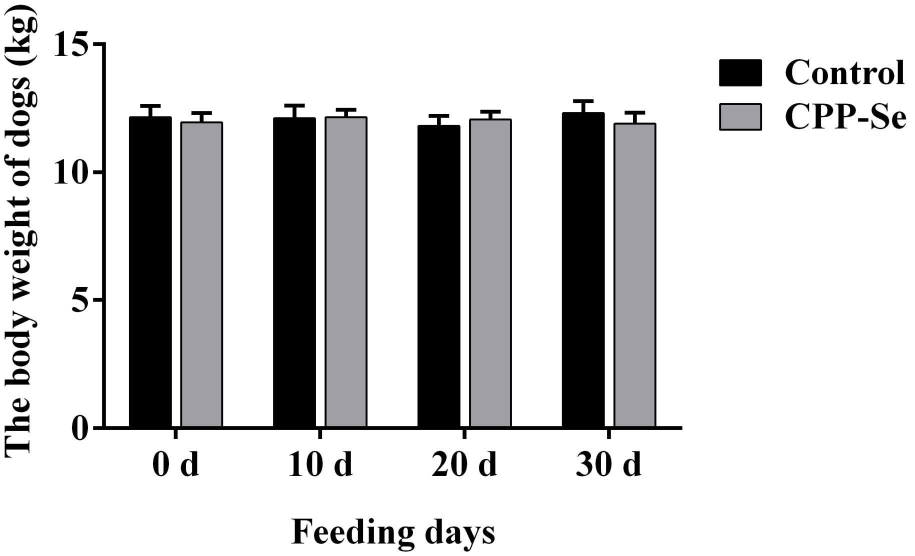

Supplementary Figure S1. The body weight of dogs during the experiment.

Supplementary Table S1. Serum biochemical data of dogs fed for 30 days.

Group	Gender	Number	Items						
			GGT (0-10 U/L)	ALT (5-125 U/L)	AST (0-50 U/L)	TB (0-15 μmol/L)	SUN (2.5-9.6 mmol/L)	SCR (44-159 μmol/L)	PI (0.81-2.19 mmol/L)
Control	Male	1	9	19	29	1	8.04	39.1	1.54
		2	7	20	33	1	8.72	56.4	1.89
		3	5	41	50	1	7.41	90.2	2.11
		4	6	28	49	1	6.46	64.8	1.23
		5	2	46	44	1.3	6.33	66	1.79
	Female	1	2	30	49	1.9	7.48	55.9	2.13
		2	2	63	33	1	9.5	62.9	1.68
		3	5	35	32	3.5	8.26	80.2	1.67
		4	5	29	38	1	6.52	52.9	0.78
		5	7	42	25	1.4	6.14	45.4	1.56
CPP-Se	Male	1	2	27	39	4.3	6.01	78.8	1.57
		2	5	23	43	1	4.47	48.2	1.55
		3	2	43	44	2.4	6.45	60.1	1.35
		4	5	31	44	2.3	7.04	72.7	1.57
		5	6	40	36	2.2	7.45	66.4	1.61
	Female	1	9	98	44	3.6	8.66	64.5	1.2
		2	2	51	30	1.6	9.5	52	1.48
		3	2	55	34	4.3	5.6	70.3	1.5
		4	6	53	27	3.8	4.61	53.3	1.47
		5	2	77	36	2.7	6.82	54.5	1.77

Note: GGT, ALT, AST, TB, SUN, SCR and PI represent glutamyltransferase, alanine aminotransferase, aspartate aminotransferase, total bilirubin, serum urea nitrogen, serum creatinine

and inorganic phosphorus, respectively.
